# Tuberculosis and Housing in the West Riding

**Published:** 1923-03

**Authors:** 


### THE PUBLIC HEALTH.

### INTERVIEWS WITH LOCAL AUTHORITIES.

### TUBERCULOSIS AND HOUSING IN THE WEST RIDING.

(By our Special Commissioner.)

### Tuberculosis.

STRENUOUS efforts are being made in the West Riding in connection with the campaign against tuberculosis. The number of beds available increased from 350 in December, 1919, to 579 in December, 1921; the dispensary nurses and health visitors made 24,500 home visits in 1921; the co-operation between general practitioners and the Tuberculosis Officers is becoming very close, especially in areas where the Officer has been some time in residence and has obtained the confidence of the practitioners. In regard to institutional treatment, the waiting list of female patients and of children is very large, and, on the other hand, there is a large number of premature and irregular discharges from the institutions, about 40 per cent. of all discharges being on account of financial difficulties at home and other economic stringencies. This has emphasised the need for Care and After-Care Committees, and arrangements have been made whereby the Joint Council of the British Red Cross Society and the Order of St. John have become associated with the West Riding Public Health Committee in certain areas, with the object of securing as far as possible that patients under home conditions are enabled to carry out the recommendations of the Tuberculosis Officers, and, also of providing for the wants of necessitous patients. The School Medical Inspectors, too, send all cases of tuberculosis and suspected tuberculosis to the dispensaries for diagnosis and treatment, and consultations between the two groups of officers frequently take place. In sending cases for institutional treatment, attention is as far as possible concentrated on early cases susceptible of a good chance of recovery. And a beginning has been made in obtaining colony treatment and training facilities in some cases. The West Riding Health Committee are, nevertheless, feeling the need for a general inquiry into the whole question of the trend and effectiveness of expenditure on the treatment, as distinct from the prevention, of tuberculosis. They see increasing and unsatisfied demands for prolonged treatment and a stationary death rate, so far as tuberculosis is concerned. It must be frankly recognised that here, as elsewhere, fresh cases of tuberculosis are being manufactured, owing mainly to the grave housing conditions, at least as fast as the cases of persons suffering from the disease are ceasing to make demands on the available forms of treatment either by death or restoration to working capacity. A resolution of the Committee was, in fact, under consideration at the date of our interview, urging the need for full and frank inquiry. The question of prevention has not, it is felt, had its proper share in the campaign against tuberculosis.

### Dirty Milk.

During the last two years Dr. Kaye's department has completed the survey of 847 cowsheds—about one-tenth of the total in the Riding—and made a comparison with the requirements of the Board of Agriculture and Fisheries. No less than 54 per cent. of the cowsheds fell short of the minimum requirements as to cubic feet of space; 74 per cent. failed as regards the standard for ventilation; 72 per cent. were insufficiently lit—20 per cent., indeed, being declared to be without light; and 64 per cent. unsatisfactory judged by any reasonable standard of cleanliness. These results confirm all past experience and show that an urgent need exists for a great improvement in the sanitary construction of cowsheds (many of the buildings in use to-day were never intended for cowsheds, and cannot be made fit for the purpose), and for a better inspection of cowsheds to secure greater cleanliness; also careful inspection of the cows for the detection of unhealthy animals, especially those suffering from general tuberculosis or tuberculosis of the udder. In 1906 the County Boroughs of Bradford, Leeds, Rotherham and Sheffield, and the County Councils of the East and West Ridings, caused a joint investigation to be made into the causes and sources of the contamination of milk, which showed that the greatest amount of contamination took place at the farm. As a result of this inquiry leaflets were prepared and distributed to farmers and dairymen, retail milk sellers, and householders in the county area. Notwithstanding all the efforts which have been made, dirty milk is still being produced. Whilst some cowkeepers show an earnest desire to produce clean milk, the apathy shown by others is amazing, and quotations from some of the replies to inquiries are interesting. One replied, "A little manure in the milk does not matter. It cleans the babies' stomachs out and does them good." Another said, "It does not matter about manure dropping in the milk, because all the milk is strained." One stated that he had heard of "horses" being groomed, but ridiculed the idea of grooming cows. The Milk and Dairies (Amendment) Act, 1922, does not help forward improvement in cowsheds, and it is not at all surprising that Health Committees with such startling facts before them feel grave concern at the apparent tenderness of the Legislature towards the farmer.

### The Grave Scarcity of Houses.

Here is one direction in which new cases of tuberculosis are being manufactured while the attempt is being made to patch up patients who have already contracted the disease. We learned that the scarcity of houses in the West Riding is becoming more and more serious owing to the inability to overcome even the demands of the growing population apart from accumulated arrears. In 1919 it was estimated that 40,000 houses were required, plus the average ordinary yearly increase of

5,000 houses before the war (a figure which was insufficient to meet the increase in population), making 10,000 for the two years 1920 and 1921, showing a total need of 50,000. Towards these, at the end of 1921, there were 10,906 houses for which tenders had been approved by the Ministry of Health for local authorities and public utility societies, so that approximately the net number of houses still needed was 39,000; in other words, it may be assumed from these facts that some 160,000 people are living under conditions of overcrowding and unhealthiness in the West Riding. There is a danger sometimes that the magnitude of the figures for a large area may fail to give an adequate picture of the distressing conditions behind them. Let us therefore localise the matter and quote from a report of the position in the Sowerby Bridge Urban District. In 1919 the house-to-house survey which was made by the District Council for the purpose of a return to the Housing Commission revealed the following position:—2,946 houses, of which 1,579 were back-to-back; 143 one-roomed houses; 495 two-roomed houses; 886 three-roomed houses; 801 four-roomed houses; total, 2,325 houses with four or less rooms, equal to 78·9 per cent. of the houses in the district. The survey also showed that overcrowded conditions existed as follows:—203 overcrowded houses on R.G.'s standard; 26 overcrowded houses had each two families; 140 unfit houses were each occupied by two families; 722 houses were unfit for habitation and not repairable; 336 unfit houses were repairable; there were three unhealthy areas affecting 563 houses, and the estimated number of houses needed was 862. Even this was found to be an under-statement of the needs of the district on a re-survey by the County Authority.

### The Drop in the Ocean.

What has been done towards remedying this grave position? From an answer given in the House of Commons to Mr. Ben Turner on December 13 we learn that the local authority have proceeded with a scheme for 43 houses with Exchequer assistance, and that the Minister of Health would be willing to give favourable consideration to any application the local authority may make for sanction to a loan for the erection of houses in the district in accordance with their general powers under the Housing Acts. Forty-three houses out of 862, or 5 per cent.! The County certainly needs wider powers—whether in the direction of lending money, raised on the security of the County rate, to employers, building societies, or to anyone else with the courage to get on with the building needed, or to step in and build themselves, Mr. Turner and his colleagues are not prepared to say until at least the intentions of the Government are indicated. We urged, in effect, that no Yorkshire-man of the true breed ever asks another man to do for him what he can do for himself, and that it has been claimed that no county in England owes less to outside help and influence than this West Riding of Yorkshire. The considered answer of the Chairman of the Health Committee and his colleagues to this suggestion is not available. But it is hardly necessary to say that the interests of the West Riding could not be in better hands, and that Mr. Ben Turner and his colleagues do not intend to let things rest where they are.

